# The Effects and Mechanisms of *Periplaneta americana* Extract Reversal of Multi-Drug Resistance in BEL-7402/5-FU Cells

**DOI:** 10.3390/molecules21070852

**Published:** 2016-06-28

**Authors:** Falu Yuan, Junyong Liu, Tingting Qiao, Ting Li, Qi Shen, Fang Peng

**Affiliations:** 1 College of Pharmacy and Chemistry, Dali University, Dali 671000, Yunnan, China; 15087290445@163.com (F.L.Y.); jonathan1253@163.com (J.Y.L.); qtt@163.com (T.T.Q.); liting0218@126.com (T.L.); 2 College of Pharmacy, Shanghai Jiaotong University, Shanghai 200240, China; qshen@sjtu.edu.cn; 3 Yunnan Provincial Key Laboratory of Entomological Biopharmaceutical R & D, Dali University, Dali 671000, Yunnan, China

**Keywords:** *P. americana*, reversion, hepatocellular carcinoma, multidrug resistance

## Abstract

The present study reports the reversing effects of extracts from *P. americana* on multidrug resistance of BEL-7402/5-FU cells, as well as a preliminary investigation on their mechanism of action. A methylthiazolyl tetrazolium (MTT) method was applied to determine the multidrug resistance of BEL-7402/5-FU, while an intracellular drug accumulation assay was used to evaluate the effects of a column chromatography extract (PACC) and defatted extract (PADF) from *P. americana* on reversing multi-drug resistance. BEL-7402/5-FU reflected high resistance to 5-FU; PACC and PADF could promote drug accumulation in BEL-7402/5-FU cells, among which PADF was more effective than PACC. Moreover, results from the immunocytochemical method showed that PACC and PADF could downregulate the expression of drug resistance-associated proteins (P-gp, MRP, LRP); PACC and PADF had no effects on the expression of multidrug resistance-associated enzymes (GST-π), but PACC could increase the expression of multidrug resistance-associated enzymes (PKC). Results of real-time fluorescence quantitative PCR revealed that PACC and PADF were able to markedly inhibit the expression of multidrug resistance-associated genes (MDR1, LRP and MRP1); PACC presented a significant impact on the gene expression of multidrug resistance-associated enzymes, which increased the gene expression of GST-π and PKC. However, PADF had little impact on the expression of multidrug resistance-associated enzymes. These results demonstrated that PACC and PADF extracted from *P. americana* could effectively reverse MDR in BEL-7402/5-FU cells, whose mechanism was to inhibit the expression of P-gp, MRP, and LRP, and that PADF was more effective in the reversal of MDR than did PACC. In addition, some of extracts from *P. americana* altered (sometimes increasing) the expression of multidrug resistance-associated enzymes.

## 1. Introduction

Hepatocellular carcinoma (HCC), which has the features of high degradation, high recurrence, and metastasis rates and whose mortality ranks third among global cancers, is the seventh most common malignant tumor type [1,2]. There are about 500 thousands new cases each year in the world, and 50% of them occur in China, with an yearly upward trend [3,4]. Although chemotherapy, such as the administration of 5-fluorouracil (5-FU), plays a critical role in the therapeutic protocol for HCC. The effectiveness of clinical first-line drugs is severely blocked and even fails due to drug resistance. Multidrug resistance (MDR) refers to the fact that tumor cells can develop resistance against multiple antitumor drugs during the process of receiving chemotherapy [5,6]. Resistance to chemotherapeutic drugs is a major barrier in the treatment of HCC [7,8], therefore, reversing MDR is deemed to be the key to improving the efficacy of chemotherapy [9]. In recent years, selecting active components with low toxicity and high efficiency from natural medicines that can reverse MDR has attracted increasing attention in the field of HCC research [10].

Researchers have disclosed that some active components found in traditional Chinese medicines (TCMs), such as psoralen, matrine, tetrandrine, and elemene, were related to the reversion of MDR, whose mechanisms of action have required further exploration [11]. Insects, the largest biological groups in Nature and with a unique immune system, are included in many TCM prescriptions. However, only a few studies have focused on the reversal of tumor MDR via insect-origin medicines. *P. americana* (Blattodea: Blattidae), commonly known as “cockroaches”, is the medicine material of three single TCMs for the treatment of wound-healing, hepatitis, and heart failure in China [12]. The medicinal efficacy of cockroaches was found thousand years ago and was recorded in ancient Chinese pharmacopeias such as *Sheng Nong’s Herbal Classic* [13] and *Compendium of Materia Medica*. Though *P. americana* is also applied as an anti-tumor remedy by local minorities in Southwestern China, studies on its efficacy in reversing MDR are rare.

Therefore, the aim of the present study was to investigate the reversing effects of extracts from *P. americana* in the HCC MDR cell line of BEL-7402/5-FU and to explore their mechanisms of action by measuring the expression of multidrug resistance-associated proteins and genes.

## 2. Results

### 2.1. BEL-7402 and BEL-7402/5-FU Cells Have Different Growth Curves

**Figure 1:**
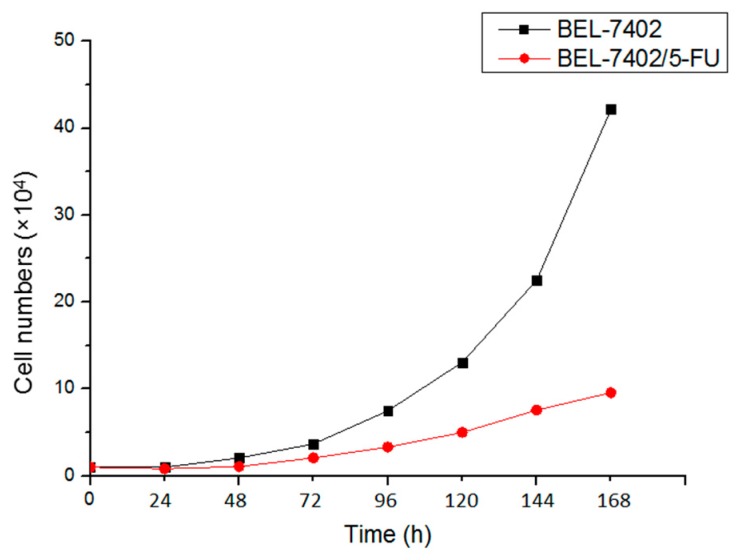
Doubling time of BEL-7402 and BEL-7402/5-FU cells.

As shown in Figure 1, it could be seen that the doubling time of BEL-7402/5-FU (51.53 h) was significantly longer than BEL-7402 cells (31.12 h). It showed that the multiplication rate of BEL-7402, promoting the initiation and progress of HCC more easily, was faster than BEL-7402/5-FU.

### 2.2. BEL-7402 and BEL-7402/5-FU Cells Have Different Sensitivity to Some Chemotherapeutic Drugs

**Figure 2:**
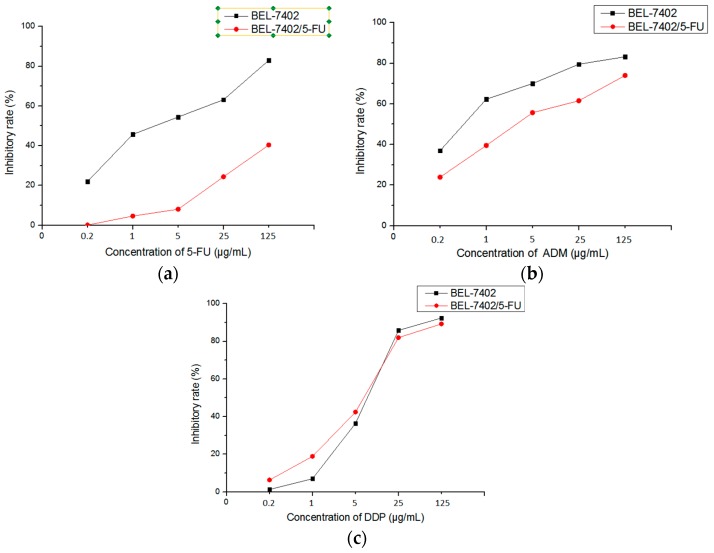
The inhibitory effect of chemotherapeutic drugs on BEL-7402 cells and BEL-7402/5-FU cells. (**a**) 5-FU; (**b**) ADM; (**c**) DDP.

IC_50_ (Table 1) of 5-FU, ADM, and DDP on BEL-7402 cells and BEL-7402/5-FU cells was obtained from the inhibitory rate (Figure 2a–c). Compared with BEL-7402 cells, BEL-7402/5-FU cells showed various degrees of drug resistance to different chemotherapeutic drugs. Among them, BEL-7402/5-FU cells performed strong resistance to 5-FU (RI, 72.81) while presenting weak drug resistance to DDP (RI, 0.82). At the same time, BEL-7402/5-FU cells demonstrated cross-resistance to ADM (RI, 8.69).

### 2.3. The PACC and PADF Extracts Have Similar Cytotoxicity in BEL-7402/5-FU Cells

**Figure 3:**
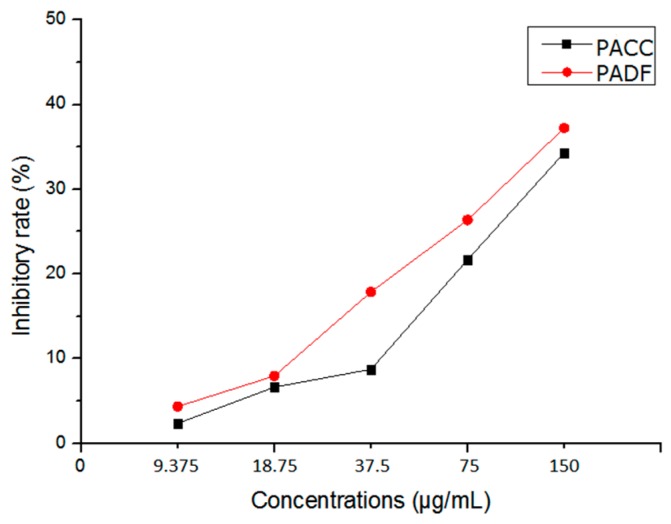
The cytoxicity of PACC and PADF on the proliferation of BEL-7402/5-FU.

PACC and PADF dose-dependently inhibited the proliferation of BEL-7402/5-FU cells, and the inhibitory rate increased as concentration rose (Figure 3). When the doses were IC_10_ and IC_5_, PACC and PADF presented weak or little toxicity to the cells. Consequently, IC_10_ and IC_5_ (Table 2) were selected as reversal doses.

### 2.4. The PACC and PADF Extracts Increase the Intracellular Accumulation of ADM

**Figure 4:**
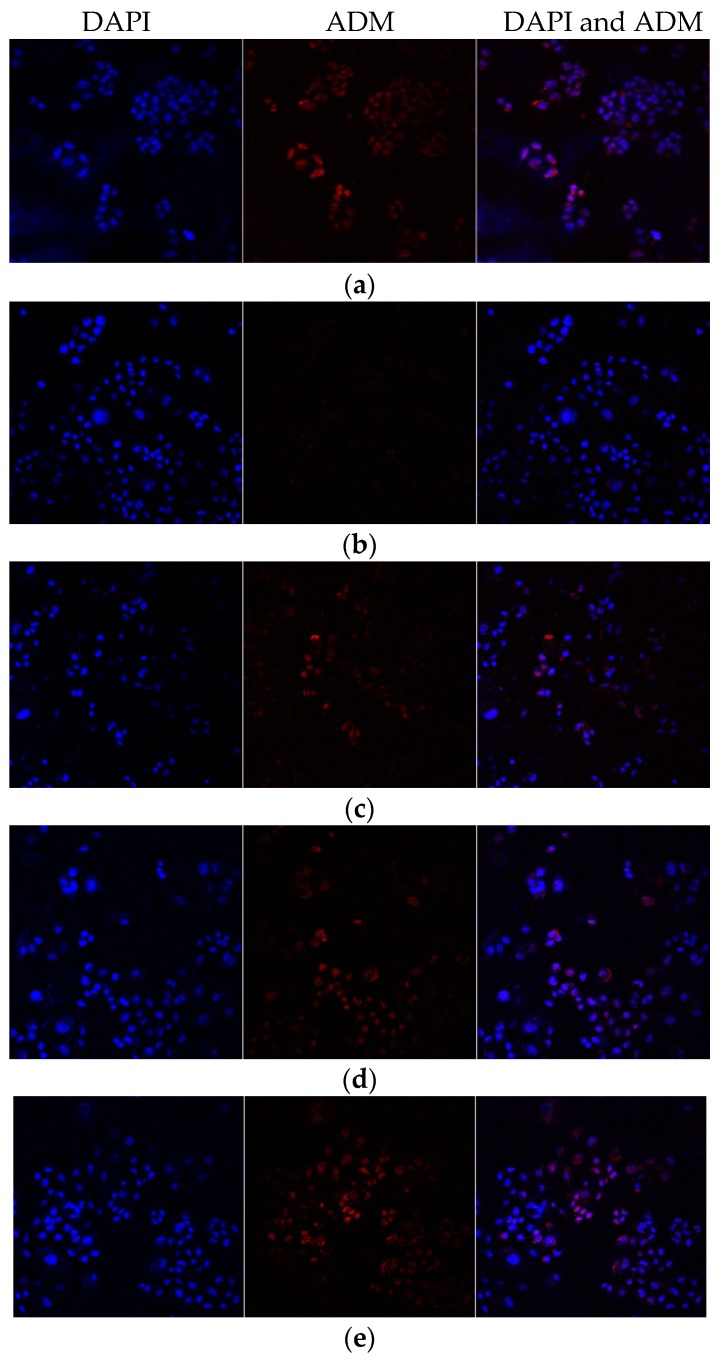

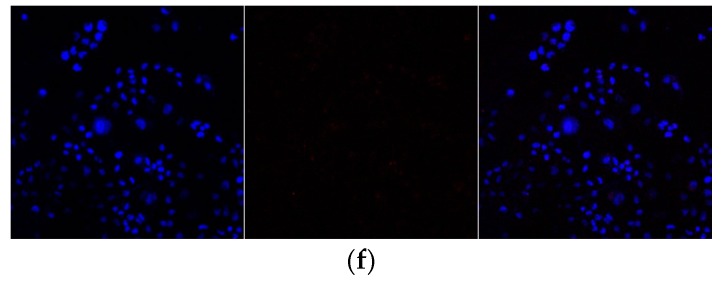
The effects of PACC and PADF on the accumulation of ADM in BEL-7402/5-FU cells (×400). (**a**) BEL-7402 cells, (**b**) BEL-7402/5-FU cells, and (**c**) BEL-7402/5-FU cells were treated with 50 μg/mL PACC; (**d**) BEL-7402/5-FU cells were treated with 85 μg/mL PACC; (**e**) BEL-7402/5-FU cells were treated with 23 μg/mL PADF; (**f**) BEL-7402/5-FU cells were treated with 45 μg/mL PADF.

In Figure 4, which was a confocal laser scanning microimage of ADM in BEL-7402/5-FU cells incubated with PACC and PADF, red fluorescence represented ADM while blue fluorescence represented DAPI. It could be seen that the amounts of red fluorescence in the BEL-7402 cells (Figure 4a) were significantly greater than those in the BEL-7402/5-FU cells (Figure 4b). Furthermore, the drug-resistant cells demonstrated cross-resistance to ADM; At the same time, the red fluorescence of the cells significantly increased when BEL-7402/5-FU cells were treated with PACC and PADF (Figure 4c–f). It would seem that PADF allowed more ADM accumulation (Figure 4c) than did PACC (Figure 4f). Moreover, PADF had a better and stronger effect than PACC.

### 2.5. The BEL-7402/5-FU Cells Have Higher Expression of Multidrug Resistance-Associated Genes Than Their Drug-sensitive Counterpart Cells

**Figure 5:**
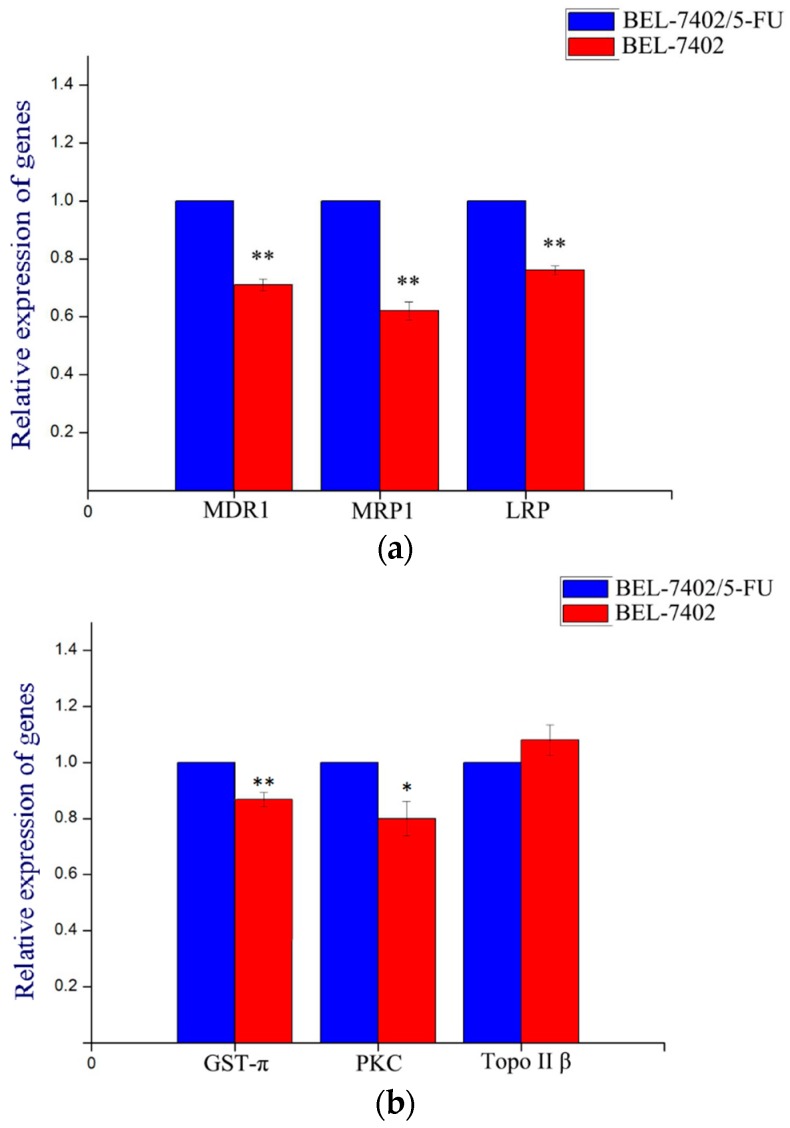
The relative expression of multidrug resistance-associated genes in BEL-7402 cells and BEL-7402/5-FU cells. Values are presented by mean ± SD (*n* = 6). (**a**) Gene expression of multidrug resistance-associated proteins; (**b**) gene expression of multidrug resistance-associated enzymes. * *p* < 0.05, ** *p* < 0.01 vs. BEL-7402.

In order to evaluate the levels of multidrug resistance-associated genes in BEL-7402/5-FU cells, we carried out a real-time fluorescence quantitative PCR assay. Results demonstrated that differences in the expressions of MDR1, MRP1, LRP (Figure 5a), GST-π, and PKC (Figure 5b) between BEL-7402 cells and BEL-7402/5-FU cells were statistically significant, while there was no difference in the expression of Topo II (Figure 5b). Therefore, the MDR of the cells was related to the high expressions of MDR1, MRP1, LRP, GST-π, and PKC. Therefore, further studies were conducted to verify if the extracts had an effect on the gene expression of MDR1, MRP1, LRP, GST, and PKC in BEL-7402/5-FU cells.

### 2.6. The PACC and PADF extRacts Inhibit P-gp, MRP, and LRP Protein Expression in BEL-7402/5-FU Cells

**Figure 6:**
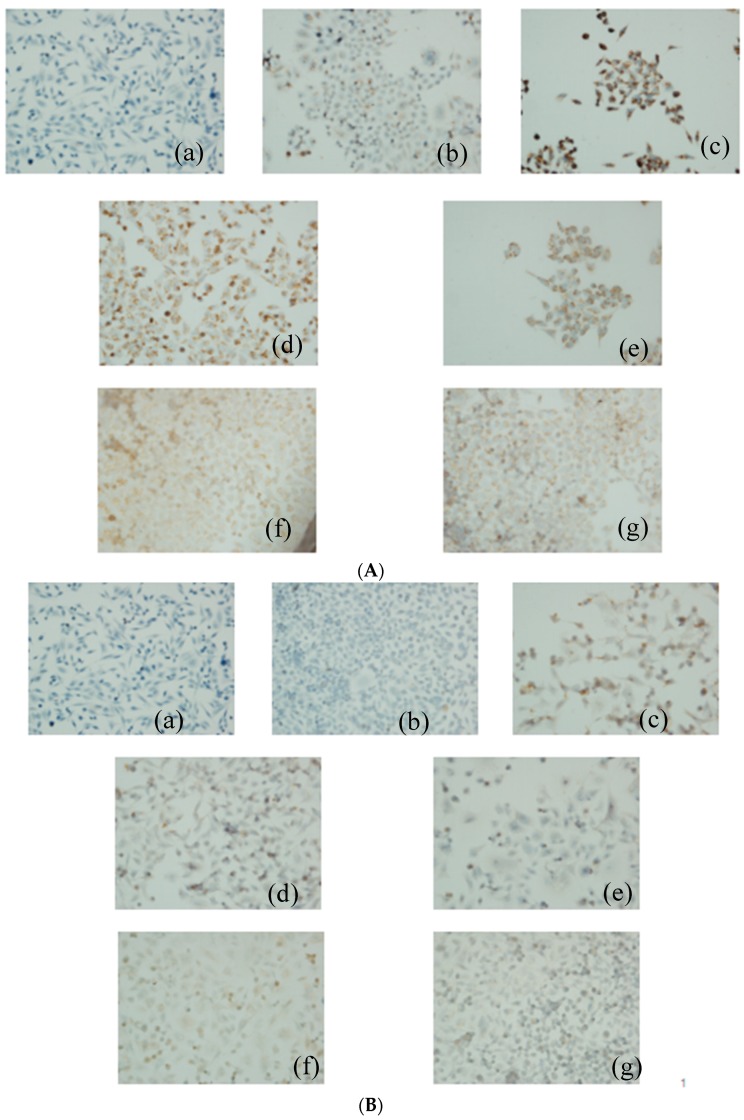

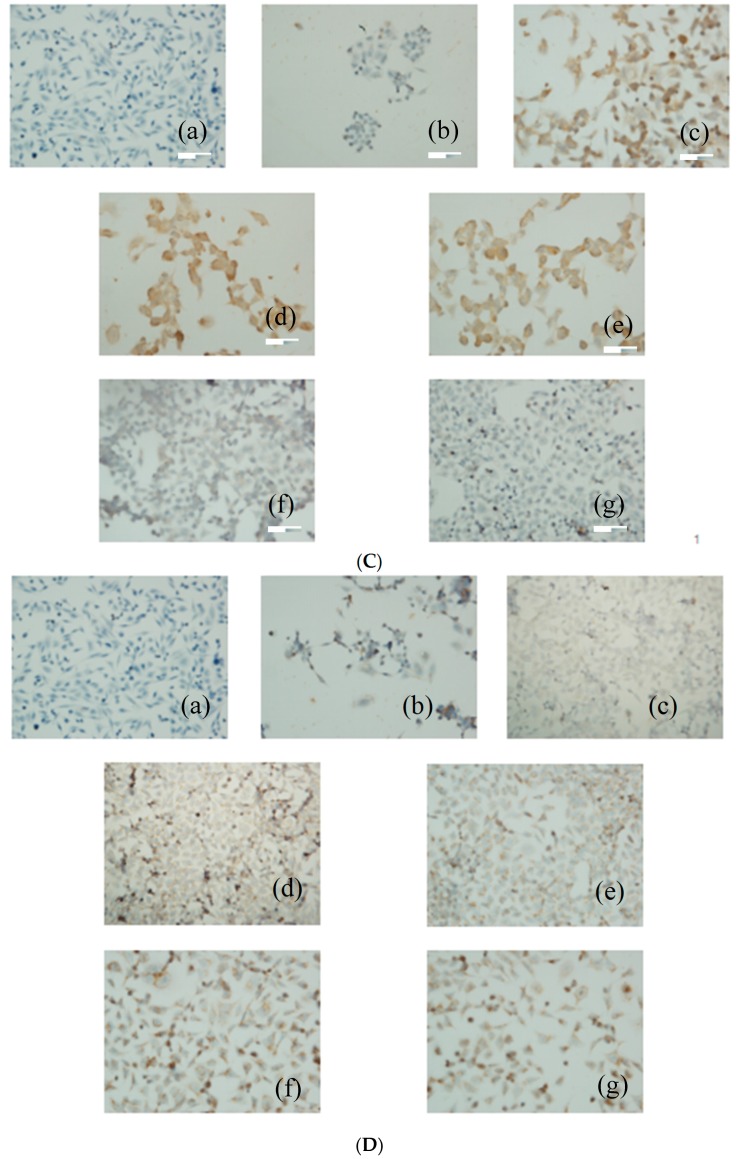

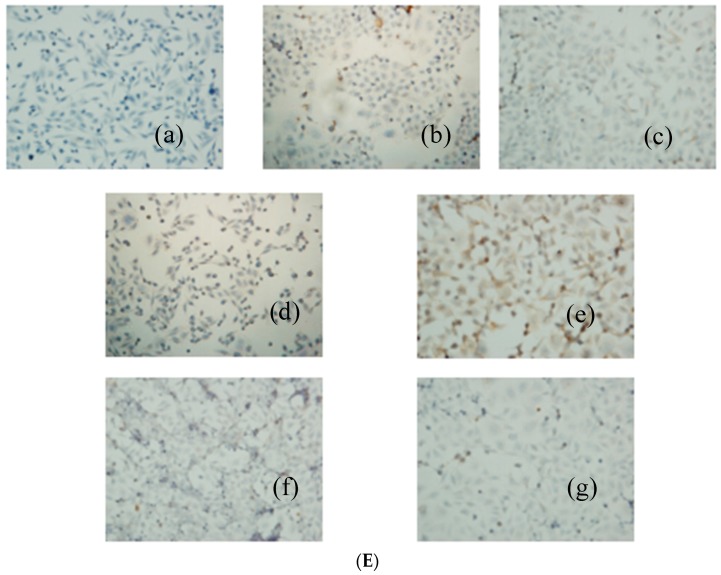
The Effects of PACC and PADF on the expression of multidrug resistance-associated proteins (×400). (**A**) Expression of P-gp; (**B**) expression of MRP; (**C**) expression of LRP; (**D**) expression of GST-π; (**E**) expression of PKC. (a) blank control group, (b) BEL-7402 cells, (c) BEL-7402/5-FU cells, and (d) BEL-7402/5-FU cells were treated with 50 μg/mL PACC; (e) BEL-7402/5-FU cells were treated with 85 μg/mL PACC; (f) BEL-7402/5-FU cells were treated with 23 μg/mL PADF; (g) BEL-7402/5-FU cells were treated with 45 μg/mL PADF.

As the immunocytochemical staining results (Figure 6) showed, PACC and PADF markedly inhibited protein the expression of P-gp, MRP, and LRP (Figure 6A–C) in BEL-7402/5-FU cells, whose inhibitory actions were positive correlated with concentrations. Brown represents the expression of proteins (the color depth indicates the expression amounts of the proteins). However, PACC and PADF had no effects on the expression of GST-π (Figure 6D), but PACC increased the expression of PKC (Figure 6E) in BEL-7402/5-FU cells while PADF had no effect.

### 2.7. The PACC and PADF Extracts Reduced the Expression of Some Multidrug Resistance-associated Genes and Altered the Expression of Some Multidrug Resistance-associated Enzymes

**Figure 7:**
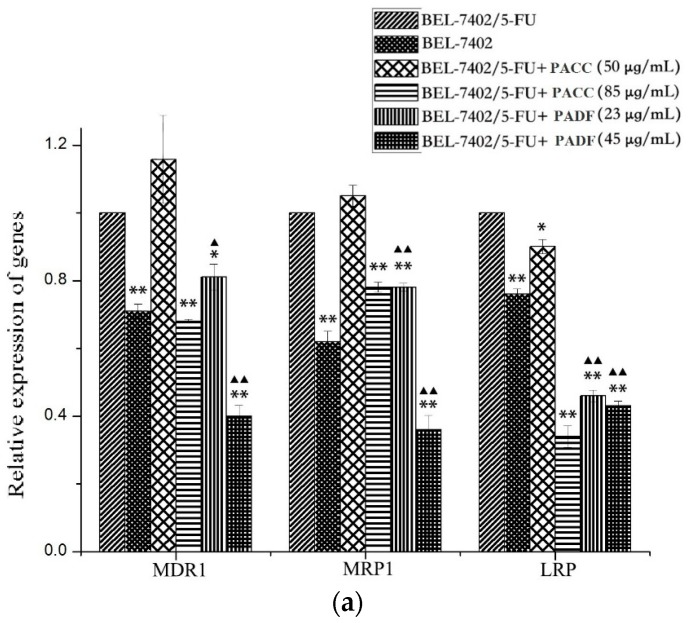

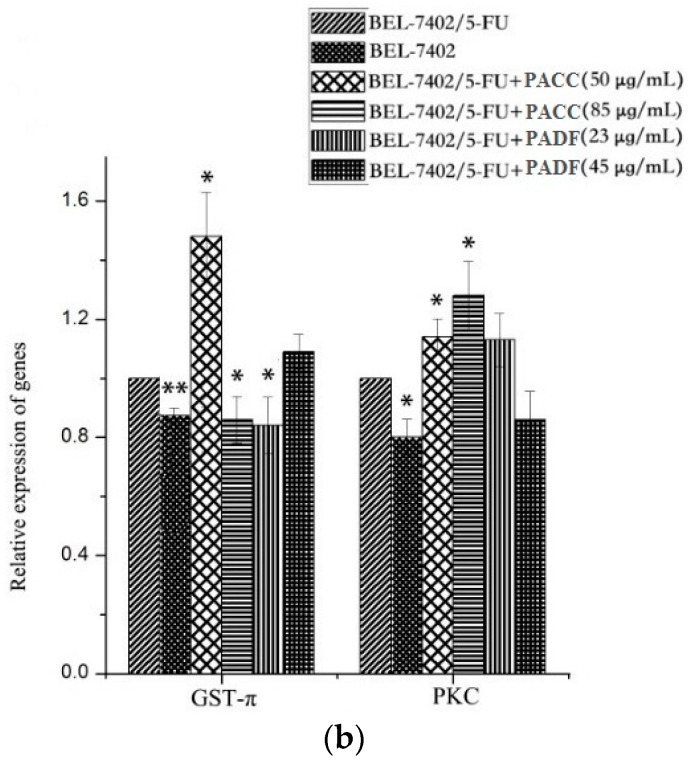
The relative expression of PACC and PADF on multidrug resistance-associated genes; Values are presented by mean ± SD (*n* = 6). (**a**) Effects of PACC and PADF from *P. americana* on the expression of multidrug resistance-associated genes; (**b**) Effects of PACC and PADF from *P. americana* on the gene expression of multidrug resistance-associated enzymes. * *p* < 0.05, ** *p* < 0.01 vs. BEL-7402, ^▲^ *p* < 0.05, ^▲▲^ *p* < 0.01 vs. PACC.

There were obvious differences (Figure 7a) in the expression of multidrug resistance-associated genes such as the MDR1, MRP1, and LRP genes between BEL-7402 cells and BEL-7402/5-FU cells, and the differences were statistically significant.

Furthermore, it could be concluded that the inhibitory effects of PADF on the expression of multidrug resistance-associated genes were statistically stronger than those of PACC. Meanwhile, PACC had a significant impact on the gene expression of multidrug resistance-associated enzymes (Figure 7b). Two doses of PACC increased the expression of PKC, and a low dose increased the expression of GST-π. However, PADF (Figure 7b) caused little impact on the expression of multidrug resistance-associated enzymes, and differences were not statistically significant.

## 3. Discussion

The mechanisms of MDR are very complex, and the main molecule-biological mechanisms of MDR in tumor cells were as follows: (1) drug efflux mediated by transport proteins [14,15], such as MRP, LRP, BCRP, and P-gp; (2) MDR mediated by enzymes [16], including topoisomerase (TOPO), glutathione-*S*-transferase (GST), and protein kinase C (PKC); (3) MDR mediated by apoptosis-associated genes [17], such as the Bcl-2 gene family, c-myc, p53, and others; (4) DNA damage repair [18]. Among them, the most important cause of MDR is drug efflux mediated by transmembrane transporter, resulting from the over-expression of multidrug resistance-associated protein and genes [19,20].

In this study, BEL-7402 cells and BEL-7402/5-FU cells were selected as a counterpart pair of drug sensitive and drug-resistant cells. From the results of the cell growth curve, we know that the growth cycle of BEL-7402/5-FU cells, compared with that of BEL-7402 cells, is significantly prolonged. Sensitivity to chemotherapeutic drugs, which provides viable and practical conditions for the intracellular accumulation of the drug assay, indicates that BEL-7402/5-FU cells have strong resistance to 5-FU and perform cross-resistance to ADM. The intracellular accumulation assay is used to confirm whether the PACC and PADF extracted from *P. americana* can reverse multidrug resistance in tumors. Results also indicated that PACC and PADF extracted from *P. americana* can promote ADM accumulation in BEL-7402/5-FU cells, whose mechanism is to reduce the efflux of chemotherapeutic drugs to reverse MDR.

The protein and gene expressions of P-gp, MRP, LRP, GST-π, and PKC in BEL-7402/5-FU cells were analyzed by an immunocytochemical method and real-time fluorescence quantitative PCR. It can be inferred that PACC and PADF reverse MDR, likely by downregulating the protein and gene expressions of P-gp, MRP, and LRP to inhibit the protein function and decrease drug efflux from cells. However, it is difficult to profoundly explore the effects and mechanisms because the multidrug resistance-associated enzyme system is complex. On the other hand, the observed were most statistically significant, possibly because the tested extracts (PACC and PADF) contained a mixture of several compounds. Thus, PACC and PADF should be separated and purified for further study. Meanwhile, although both PACC and PADF could reverse MDR to some degree, the effects of PADF on reversing MDR are better and stronger than PACC under non-toxic doses for cells, and the extraction process of PACC is more complicated. Consequently, PADF, being more effective and easier to extract, might be further studied in order to isolate compounds to reverse MDR phenotype of cancer cells.

To date, there are two measures for reversing MDR in the clinical treatments of tumors: (1) the use of chemotherapeutic drugs, which, when combined with reversal agents, resist MDR, and (2) the use of drugs to which patients are sensitive, or an increased dosage. The latter method is widely used, but the effect is not ideal. Although the present study shows that PACC and PADF can reverse MDR in BEL-7402/5-FU cells, the mechanism here clarified could only be justified by the protein and gene expressions of P-gp, MRP, and LRP; thus, further studies about drug resistance-associated enzyme systems are still needed to provide an experimental and theoretical basis to overcome MDR and to improve the efficacy of the combination of PACC and PADF with chemotherapeutic drugs in HCC cells. In addition, mechanisms of MDR are very complex in tumors as a result of the combined action of multiple genes and factors. In the future, further studies attempting to verify whether there are other mechanisms of PACC and PADF in reversing MDR should be continued. At the same time, in vivo experiments to confirm the reversal effects of PACC and PADF are also necessary.

## 4. Materials and Methods

### 4.1. Cell Lines and Test Drugs

Human hepatocellular carcinoma BEL-7402 cells and multi-drug resistant human hepatocellular carcinoma cell line BEL-7402/5-FU cells were obtained from Shanghai Meixuan Biological Technology Co., Ltd. (Shanghai, China). Bioactive extracts of *P. americana*, PACC and PADF, were prepared and kindly provided by Dr. Cheng-Gui Zhang and his coworkers at the National–Local Joint Engineering Research Center of Entomoceutics, Dali University (Dali, China).

The extraction method of PADF (fulvous and viscous cream) is as follows: the *P. americana* polypides, after the drying process, were crushed and cold-soak-extracted three times with 70% alcohol. Each time, the extracted liquid was filtered, and the filtered fluid was merged together and concentrated. Ultimately, an extract was prepared for the experiments [21].

The extraction method of PACC (freeze-dried powder) is as follows: the resulting extract above was added to a polyamide column and eluted with water and different proportions of aqueous methanol, collected quantitatively, and concentrated separately. One stroke segment after freeze-drying was used as a sample for the experiments [22].

### 4.2. Chemicals and Reagents

RPMI-1640 medium was bought from Hyclone (Logan, NM, USA) and fetal bovine serum from Gibco (California, CA, USA); a mixture of penicillin and streptomycin, 0.25% Trypsin-EDTA, dimethyl sulfoxide (DMSO), and methylthiazolyl tetrazolium (MTT) were all obtained from Solarbio Co. (Beijing, China) 5-FU was bought from Tianjin Kingyork Amino Acid Co. (Tianjin, China); adriamycin (ADM) was bought from Shanxi Pude Pharmaceutical Co. (Datong, China); cispaltin (DDP) was bought from Qilu Pharmaceutical Co. (Jinan, China) Fluorescence quantitative PCR kits were provided by Shanghai Solmon Biotechnology Co. (Shanghai, China), Ltd, and primers were achieved from Shanghai Sangon Biotechnology Co. (Shanghai, China). immunohistochemical staining kits were provided from Wuhan Boster Biological Co. (Wuhan, China).

### 4.3. Cell Culture

BEL-7402 cells were regularly cultured in RPMI-1640 medium containing 10% fetal bovine serum at 37 °C in 5% CO_2_ incubator, then the medium was changed every 2 days and the next generation was produced after culturing for 3–4 days [23]. BEL-7402/5-FU cells were cultured in the aforementioned medium with 20 μg/mL 5-FU under the same conditions to keep the drug resistance for at least 1 week before the experiments [24]. Experiments were performed on the cells in logarithmic growth phase.

### 4.4. Determination of Growth Curve

BEL-7402 cells and BEL-7402/5-FU cells of logarithmic growth phase were digested by 0.25% Trypsin-EDTA and seeded in a 24-well plate at 1 × 10^5^ cells per milliliter (with 100 μL cell suspension and 900 μL medium) for 24 h. Every 24 h, three wells were randomly selected and digested to prepare single-cell suspension for cell counting and a drawing growth curve. The Patterson method [23] was applied to measure the doubling time of cells.

### 4.5. Determination of Sensitivity of Cells to Chemotherapeutic Drugs

BEL-7402 cells and BEL-7402/5-FU cells in logarithmic growth phase were digested with 0.25% Trypsin-EDTA and seeded in a 96-well plate at 2 × 10^4^ cells per well with 100 μL for culturing 12 h. A range of concentrations of 5-FU, DDP, and ADM (0.2, 1, 5, 25, 125 μg/mL) were added to designated wells with 100 μL per well. Meanwhile, the blank control group only contained a medium and a negative control group without drugs, which only contained cells that were set for experiments. Each concentration was repeated in 6 wells. After 48 h, the cells were incubated for 4 h following the addition of 20 μL of 5 mg/mL 3-(4,5-dimethylthiazol-2-yl)-2,5-diphenyltetrazolium bromide (MTT), the old medium was then abandoned, and 150 μL of DMSO was added to each well to measure an absorbance value at 490 nm [25]. All experiments were independently repeated three times and the average values were adopted.

### 4.6. Cytotoxicity of PACC and PADF

BEL-7402/5-FU cells of logarithmic growth phase were digested by 0.25% Trypsin-EDTA and seeded in a 96-well plate at 2 × 10^4^ cells per well with 100 μL for 12 h. A range of concentrations of PACC and PADF (9.375, 18.75, 37.5, 75, 150 μg/mL) were added to designated wells, with 100 μL per well. Meanwhile, the blank control group only contained medium and a negative control group without drugs, which also only contained cells that were set for experiments. Each concentration was repeated in 6 wells. After 48 h, the absorbance value per well was measured at 490 nm by the aforementioned MTT method, and the IR was calculated by the same formula to evaluate IC_5_ and IC_10_ value for setting the nontoxic doses for reversal experiments. IC_5_ and IC_10_ can be obtained from IR of different concentrations by Spss 17.0.

### 4.7. Design of Experimental Group

Group 1: BEL-7402 cells. Group 2: BEL-7402/5-FU cells. Group 3: BEL-7402/5-FU cells treated with 50 μg/mL PACC. Group 4: BEL-7402/5-FU cells treated with 85 μg/mL PACC. Group 5: BEL-7402/5-FU cells treated with 23 μg/mL PADF. Group 6: BEL-7402/5-FU cells treated with 45 μg/mL PADF.

### 4.8. Intracellular Accumulation of ADM Assay

BEL-7402/5-FU cells were digested and seeded in slides to prepare a round coverslip at 5 × 10^4^ cells per well for 12 h incubation. After 12 h, PACC and PADF were added to designated wells with 100 per well and incubated at 37 °C in 5% CO_2_. Drugs were added into designated wells according to the experimental groups. After 48 h, ADM was added in cells for treating 2 h. After that, cells were washed three times by cold PBS and fixed for 30 min with 4% paraformaldehyde (PFA) away from light. Then round coverslip was washed by PBS two times, stained for about 3–5 min with 1–2 drops of diamidinophenylindole (DAPI) and mounted by a mounting solution, and then observed via laser scanning confocal microscopy (LSCM).

### 4.9. Immunocytochemical Assay

Immunocytochemical assay was used to observe the effects of PACC and PADF on multidrug resistance-associated proteins in BEL-7402/5-FU cells. BEL-7402/5-FU cells were digested and seeded in slides to prepare round coverslip at 5 × 10^4^ cells per well with for 12 h incubation. PACC and PADF were added to designated wells with 100 per well and incubated at 37 °C in 5% CO_2_. Drugs were added into designated wells according to the experimental groups. Drugs were added into designated wells according to the experimental design. After 48 h, the immunocytochemical assay was used to detect multidrug resistance-associated proteins according to the operating instructions of the kits.

### 4.10. Real-Time Fluorescence Quantitative PCR Analysis Assay

A real-time fluorescence quantitative PCR assay was used to confirm the expression of multidrug resistance-associated genes and enzymes and to observe the effects of PACC and PADF on the expression of multidrug resistance-associated genes and enzymes. BEL-7402/5-FU cells were digested and seeded in a 12-well plate at 1 × 10^5^ cells per milliliter with 2 mL per well for 12 h incubation. PACC and PADF were added to designated wells at 100 μL per well based on the former experimental groups.

Drugs were added into designated wells according to the experimental design. After 48 h, RNA was extracted with a Trizol one-step method and dissolved by diethyl pyrocarbonate (DEPC). The concentration of RNA was determined with a spectrophotometer to establish a 10 μL reaction system of reverse transcription based on the operating instructions of the fluorescence quantitative PCR kits with the following conditions: pre-degenerated at 95 °C for 5 min, degenerated at 95 °C for 30 s, renaturated at 58 °C for 30 s, and extended at 72 °C for 30 s with 40 cycles. β-actin was used as an internal control for the loading. The forward and reverse PCR primers were as follows (Table 3).

### 4.11. Data Analysis Statistics

Doubling time (DT) was calculated according to the following formula (1):

DT = t × lg2/(lgN_t_ − lgN_0_) (1)

where t is time of N_t_ minus N_0_; N_0_ is the number of initial cells; N_t_ is the number of terminal cells;

The inhibitory rate (IR) was calculated according to the following formula (2):

IR (%) = (1 − A_x_/A_0_) × 100% (2)

where A_x_ is the absorbance value of the treatment group, and A_0_ is the absorbance value of the control group.

Resistance index (RI) was calculated according to the following formula (3):

RI = IC_50(R)_/IC_50(S)_ (3)

where IC_50(R)_ is the IC_50_ value of drug-resistant cell BEL-7402/5-FU, and IC_50(S)_ is the IC_50_ value of sensitive cell BEL-7402. All experiments were repeated three times independently. All experiments were repeated three times and took the average value.

IC_50_ was obtained from the following formula (4):

IC_50_ = lg^−1^{X_m_ − I × (P − (3 − P_m_ − P_n_)/4)} (4)

where X_m_ is the largest concentration, I is dilution factor, P is sum of inhibitory rate, P_m_ is the maximum IR, P_n_ is the minimum IR.

The relative expression quantity (RQ) of MDR1, MRP1, and LRP were calculated by the following formula (5):

RQ = 2^−^^△△Ct^, △△Ct = △Ct_(ts)_ − △Ct_(rs)_ (5)

where Ct is the threshold cycle, indicating the cycle number at which the amount of amplified target genes reaches the fixed threshold. Then, △Ct of the test sample and reference sample can be separately calculated by the following formula:

△Ct = Ct_(tar)_ − Ct_(hou)_ (6)

where Ct_(tar)_ is the Ct value of the target gene, while Ct_(hou)_ is the Ct value of the housekeeping gene, β-actin.

Data were expressed by mean ± SD. One-way analysis of variance (ANOVA) analysis was used to determine the differences among the study groups with SPSS v.19.0. (IBM, Chicago, IL, USA, 2010) A *p*-value of less than 0.05 was considered statistically significant.

## 5. Conclusions

In conclusion, the present study demonstrated that PACC and PADF could promote ADM accumulation in BEL-7402/5-FU cells so that the cross-resistance could be reduced to some extent, which was significant for achieving a reversal of MDR. PACC and PADF could inhibit the expression of multidrug resistance-associated proteins (P-gp, MRP, LRP) in BEL-7402/5-FU cells and also downregulate the expression of multidrug resistance-associated genes (MDR1, MRP1, LRP). These results implied that PACC and PADF reverse MDR of HCC by inhibiting the expression of multidrug resistance-associated proteins and genes. Moreover, PADF was more effective in the reversal of MDR than PACC. In addition, some of extracts from *P. americana* altered (sometimes increasing) the expression of multidrug resistance-associated enzymes.

## Figures and Tables

**Table 1:** Sensitivity of BEL-7402 cells and BEL-7402/5-FU cells to chemotherapeutic drugs.

Drugs	IC_50_ (µg/mL)		RI
	BEL-7402	BEL-7402/5-FU	
5-FU	2.787 ± 0.34	202.910 ± 1.49 **	72.81
ADM	0.529 ± 0.07	4.595 ± 0.32 *	8.69
DDP	7.379 ± 0.40	6.066 ± 0.19	0.82

Values are expressed by Mean ± SD (*n* = 6). * *p* < 0.05, ** *p* < 0.01 vs. BEL-7402.

**Table 2:** IC_5_ and IC_10_ of PACC and PADF on BEL-7402/5-FU cell ($\bar{x}$ ± s, *n* = 6).

Drugs	BEL-7402/5-FU	
	IC_5_ (µg/mL)	IC_10_ (µg/mL)
PACC	50.12 ± 3.77	84.25 ± 3.20
PADF	23.24 ± 0.74	46.03 ± 1.29

**Table 3:** List of primers [26,27].

Primers		Sequence (5′ to 3′)	Base Numbers	Purification Method
β-actin	Forward	AAGGCTGTGGGCAAGG	16	HAP *
β-actin	Reverse	TGGAGGAGTGGGTGTCG	17	HAP
MDR1	Forward	GGAGCGGTTCTACGA	15	HAP
MDR1	Reverse	ACGATGCCCAGGTGT	15	HAP
MRP1	Forward	GTCGGAACAAGTCGTGCCTG	20	HAP
MRP1	Reverse	CAAAGCCTCCACCTCCTCA	19	HAP
LRP	Forward	GGCTCCTTCCGCTACGT	17	HAP
LRP	Reverse	GCCGAGACCGCTCAATAC	18	HAP
GST-π	Forward	CTGGAAGGAGGAGGTGGTG	19	HAP
GST-π	Reverse	GACGCAGGATGGTATTGGAC	20	HAP
PKC	Forward	CCCAAACATTGACAAATCCTAACC	24	HAP
PKC	Reverse	CAACCAAGGAGGGTACCAGATG	22	HAP
Topo II β	Forward	GAAACGGAATCCTTGGTCAGAT	22	HAP
Topo II β	Reverse	TTTCGGCTGCTGCTCTCCTA	20	HAP

* HAP (High affinity purification), is a new purification method of DNA which purity is up to 98%.
